# Barriers to promoting breastfeeding in primary health care in Mexico: a qualitative perspective

**DOI:** 10.3389/fnut.2023.1278280

**Published:** 2024-01-09

**Authors:** Elizabeth Hoyos-Loya, Cecilia Pérez Navarro, Soraya Burrola-Méndez, Sonia Hernández-Cordero, Isabel Omaña-Guzmán, Matthias Sachse Aguilera, Mónica Ancira-Moreno

**Affiliations:** ^1^Observatorio Materno Infantil (OMI), Universidad Iberoamericana, Mexico City, Mexico; ^2^Health Department, Universidad Iberoamericana, Mexico City, Mexico; ^3^Research Center for Equitable Development EQUIDE, Universidad Iberoamericana, Mexico City, Mexico; ^4^Pediatric Obesity Clinic and Wellness Unit, Hospital General de México “Dr. Eduardo Liceaga”, Mexico City, Mexico; ^5^United Nations International Children’s Emergency Fund (UNICEF), Mexico City, Mexico

**Keywords:** breastfeeding, primary health care, child health services, quality of health care, postpartum, infant, prenatal care

## Abstract

**Objective:**

This article aimed to identify the main barriers related to promoting and counseling breastfeeding (BF) at the Primary Health Care (PHC) in Mexico.

**Methodology:**

A qualitative study with a phenomenological approach was carried out in 88 health centers of the Ministry of Health in the states of Chihuahua, Oaxaca, Chiapas, Veracruz, Mexico, and Yucatan. From September to November 2021, we interviewed 88 key health professionals (HPs) (physicians, nurses, nutritionists, and others) from the PHC and 80 parents of children under 5 years old. In addition, nine focus groups were conducted with parents and caregivers. The data obtained were triangulated with information from focus groups and semi-structured interviews.

**Results:**

Of the total interviews, 43.2% (*n* = 38) were nurses, 29.5% (*n* = 26) were physicians, 19.3% (*n* = 17) were nutritionists, and the rest were other health professionals. In the group of users, 97.6% (*n* = 121) were women. We identified contextual barriers, such as the lack of well-trained health professionals and the scarcest nutrition professionals, as material resources in the health units, without mentioning the low user attendance at their control consultations. Furthermore, we identified barriers related to the orientation and promotion of breastfeeding in health units, including a lack of specific strategies, ineffective communication, and the recommendations of commercial milk formulas.

**Conclusion:**

The results presented reflect the reality of Mexico in relation to BF, making it urgent to take immediate action to improve the quality of nutritional care related to the promotion and orientation of BF at the PHC.

## 1 Introduction

Breastfeeding (BF) is paramount for the optimal development of newborns. Current guidelines on infant feeding practices underscore the importance of initiating breastfeeding within the first hour of life, followed by 6 months of exclusively breastfeeding on demand and continuing breastfeeding for up to 2 years or beyond (1–3). Despite these recommendations, social and cultural stigmas persist, hindering BF’s initiation and continuity (4–6).

In response to this problem, the Mexican national health system has designated BF as a priority in national policy (7). Strategies and targeted actions have been implemented to enhance health services and promote infant development by supporting and protecting BF (8). Among these strategies is hospitals’ adherence to the Baby-Friendly Hospital Initiative (BFHI) (9, 10), which stands out as a commitment to promoting, protecting, and supporting BF. The BFHI, updated in 2018, emphasizes that healthcare institution personnel must possess the knowledge, competencies, and skills required to ensure BF support (Step 2), among other activities promoting the practice. However, a significant gap is noted as primary-level units, including health centers, are not included in this initiative (9).

Concurrently with the BFHI, the National Breastfeeding Strategy, implemented from 2014 to 2018, emerged as a political instrument to address sustainable development goals, focusing on reducing malnutrition and infant mortality (10). Due to the lack of accreditation of all medical units nationwide, a key objective of this strategy was to increase the number of children fed breast milk from birth to at least 2 years of age (10). However, its evaluation and monitoring yielded null results (11). Additionally, the dissemination and surveillance of compliance with the International Code of Marketing of Breastmilk Substitutes and the correct, rational, and medically indicated use of these products are fundamental actions to prevent discouraging BF practice, with implications for the health and economy of the health sector and families (12).

Mexico also has technical regulations to promote exclusive breastfeeding, breastfeeding techniques, and guidance on common issues through health units that focus on encouraging mothers to breastfeed (1). However, despite efforts and the increase in exclusive breastfeeding (from 14.4% in 2012 to 35.9% in 2021), the current prevalence remains considerably below priority targets for maternal and child health (13–15).

The benefits of BF have been extensively documented, ranging from combating all forms of infant malnutrition to promoting health throughout the life cycle (16). Recognized as the most cost-effective strategy to prevent infant mortality, it is estimated to prevent 13.8% of deaths in children under 2 years in medium- and low-income countries (4, 17). Moreover, BF brings economic benefits to families (18) and the health system, reducing the use of health services by preventing diseases in newborns (19, 20). Studies such as that of Hanieh et al. (20) indicate that infants exclusively breastfed at 6 weeks after birth have lower odds of hospitalization for diarrhea (OR 0.37; 95% CI 0.15, 0.88) and suspected pneumonia (OR 0.39; 95% CI 0.20, 0.75).

In addition to benefits for children, BF also provides multiple advantages for breastfeeding mothers, including contributions to their health and wellbeing, reduced risk of breast and ovarian cancers, decreased risk of type 2 diabetes, increased family and national resources, and environmental respect (21–23).

Despite these benefits and efforts, various reasons, such as the perception of low milk supply, physical issues such as nipple cracks or pain, mastitis, and maternal return to work, lead some mothers to abandon or not initiate breastfeeding (4, 5, 24). Therefore, breastfeeding is an activity that mothers cannot carry out entirely on their own; the intervention of healthcare personnel, providing counseling, group support, and willingness to support the mother are required (25–27).

In this context, it is essential for all women to receive quality care during pregnancy, childbirth, and the postnatal period (2). For this, the definition of quality of care proposed by the World Health Organization is adopted, defining it as “the set of diagnostic and therapeutic services most suitable for optimal healthcare, taking into account all patient and medical service factors and knowledge to achieve a result with the minimum risk of effects and maximum patient satisfaction” (28).

In this regard, healthcare professionals play a crucial role in guiding and promoting BF respectfully and sensitively to individual user preferences and cultural characteristics, providing healthcare services that ensure benefits for all, regardless of factors such as ethnicity, geographic location, and socioeconomic status. Additionally, care must be safe, effective, timely, and efficient, ensuring optimal quality (29).

Despite the importance of studying the provision of quality services to promote this practice from pregnancy onwards, few studies have addressed this problem in the health system. Therefore, this study aimed to identify barriers to promoting and counseling breastfeeding at Primary Health Care (PHC) in Mexico. Results from this study would guide the development of policies and interventions regarding improving the orientation and promotion of BF in first-level healthcare units, which may generate an increase in the prevalence of exclusive and continued BF in this country.

## 2 Materials and methods

We conducted a cross-sectional study with a mixed approach in the Mexican Secretary of Health first-level healthcare units to assess the quality of nutritional care during preconception, pregnancy, postpartum, childhood, and preschool age. This article presents the findings related to identified barriers to promoting and counseling breastfeeding. Data collection took place from September to November 2021 in centers affiliated with the Mexican Secretariat of Health located in the following states: Chihuahua, Oaxaca, Chiapas, Veracruz, the State of Mexico, and Yucatan.

### 2.1 Population and study unit

The study population included two groups: (a) healthcare professionals (HPs) (including nursing, medical, and nutrition staff) and (b) users [women in preconception, pregnant, postpartum, or mothers with infants (0–2 years), preschoolers (3–5), or their partners]. Unit selection for health centers involved random sampling, considering the total number of health centers in the six selected states, with 50% of the medical units having an acceptable rating, a confidence level of 95%, and a precision of 10%, aiming for representation in all six states. A sample of 97 units was estimated. Access to health centers was facilitated through state authorities and, in some cases, with the support of health authorities in the corresponding jurisdictions.

For the selection of user participants or their partners, the criteria included: (a) being a woman in preconception, (b) being pregnant, or (c) being a parent of a child under 5 years. Regarding HP, the criterion was initially to have worked in the health center for at least 2 years. However, due to frequent staff rotation, this criterion was eliminated. In both groups, participants needed to be at least 18 years old and provide signed informed consent to participate in the research. At least one interview with an HP, an interview with a user in each health center, and 30 focus groups (5 in each state) were expected to be conducted.

### 2.2 Data collection

Two instruments were used for data collection: a semi-structured interview guide and a guide for focus groups. Given the characteristics of the target population, eight semi-structured interview guides were designed: (1) physician staff, (2) nursing staff, (3) nutrition staff, (4) women in preconception, (5) pregnant women, (6) women in postpartum, (7) mothers, fathers, or caregivers of infants aged 0–2 years, and (8) mothers, fathers, or caregivers of children aged 3–5 years. The instruments were developed following a phenomenological approach (30).

For HP, the emphasis was on exploring their medical care practices, barriers, and training in quality nutritional care. In the case of users, the interviews explored perceptions of breastfeeding promotion and counseling received while using public health services. For focus groups, a guide for users was designed, specifying sections to explore and deepen according to the life stage. All instruments were previously tested, adjusted, and validated in a pilot test in five health centers in the State of Mexico.

Three trained and standardized researchers conducted the interviews and focus groups. The HP in charge of the unit received the research team in the health center. Subsequently, the researchers were directed to the HP for the interview. Regarding user interviews, quota sampling (31) was employed, with the researcher directly requesting an interview with a woman in the medical office area. Due to time constraints in the units, priority was given to conducting at least one interview with an HP and another with a user.

Focus groups were convened by health center staff 1 day in advance, requesting the presence of five to six users belonging to the same life stage (preconception, pregnancy, postpartum, and/or mothers with infants or preschoolers) in the units at 8:00 a.m. However, only nine of the expected focus groups were conducted due to low user attendance related to the COVID-19 pandemic. In Estado de México, conducting a focus group was impossible due to difficulties in gathering users.

They were conducted in a private space within the designated areas of the health center during working hours. A private, ventilated space with chairs was provided within the unit for focus groups. Data collection took place in 88 rural and urban health centers across the six states, with the participation of 88 HP and 119 users, including 39 in focus groups. All participants were asked for sociodemographic information, which were recorded with the REDCap software.

### 2.3 Data analysis

All interviews and focus groups were audio-recorded with participants’ informed consent and subsequently transcribed by the nine field researchers. Information management was consistently anonymous and confidential.

Data analysis followed grounded theory principles (32), allowing for the generation of emerging categories (open coding) added to *a priori* categories in a codebook developed by at least two researchers (see Table 1). Representative narratives were selected to achieve category saturation, aiming for a robust theoretical explanation of the phenomenon. Differences and convergences in the data among researchers were discussed. Taguette software supported information analysis (33).

**Table 1:** Breastfeeding codebook.

Actor	Category	Description
User	BF knowledge and information	Overview about the users’ knowledge and how they get it
	BF recommendations	Guidance on techniques for effective latching, breast extraction, and breast massage users receive at the health center
	BF length	Period that women say they have given exclusive breastfeeding and/or continued breastfeeding
	BF barriers	Difficulties identified by women to follow the BF recommendations during the postpartum period, the infant, and the preschool child stage
	Formula	Use or introduction of milk formula in babies reported by women
Health professionals	BF promotion	Strategies or actions to promote exclusive breastfeeding and continued breastfeeding carried out by the HP in the health center
	BF guidance	Breastfeeding information HP give to users during control medical consultation
	BF follow-up	Follow-up given to breastfeeding provided by postpartum mothers and up to 2 years of age of the child
	BF barriers	Difficulties identified by HP for women to follow the BF guidance or recommendations

BF, breastfeeding.

### 2.4 Ethical considerations

Ethical aspects of the research outlined in the Declaration of Helsinki were considered. The study received approval from the Ethics Committee of the Iberoamerican University (103/2021). The identity of participants remains anonymous, and permission to publish results was obtained through informed consent.

## 3 Results

A total of 88 interviews with HP were conducted, and 80 interviews and 9 focus groups were conducted with users in different age stages. Due to technical issues, 18 interviews were discarded, 8 from users. The mean age in HP was 40.2 years (SD 9.9). Of the total interviews, 43.2% (*n* = 38) were nurses, 29.5% (*n* = 26) were physicians, 19.3% (*n* = 17) were nutritionists, and the rest were other health professionals. In the group of users, 97.6% (*n* = 121) were women. Most users reported common law and married civil status at 47.6% and 44.4%, respectively. They had completed middle school (46%) and high school (24.2%); only 10.5% had a bachelor’s degree. Tables 2 and 3 show the main characteristics of the HP and of the users who participated in the different techniques. The main barriers identified in the research are presented in three sections: contextual barriers, barriers from users’ perspectives, and barriers from health professionals.

**Table 2:** Sociodemographic characteristics of health professionals.

	Chiapas *n* = 14	Chihuahua *n* = 13	State of Mexico *n* = 20	Oaxaca *n* = 11	Veracruz *n* = 18	Yucatan *n* = 12	Total *n* = 88
**Age (years)**							
Media (DE)	40.5 (7.4)	37.4 (7.4)	38.3 (9.6)	41.9 (9.4)	43.6 (8.8)	39.7 (16.1)	40.2 (9.9)
**Sex *n* (%)**							
Woman	9 (64.3)	10 (76.9)	16 (80)	9 (81.8)	15 (83.3)	6 (50)	65 (73.9)
Man	5 (35.7)	3 (23.1)	4 (20)	2 (18.2)	3 (16.7)	6 (50)	23 (26.1)
**Marital status *n* (%)**							
Single	3 (21.4)	5 (38.4)	5 (25)	4 (36.4)	7 (38.9)	5 (41.7)	29 (33)
Married	10 (71.3)	4 (30.8)	13 (65)	3 (27.3)	6 (33.3)	6 (50)	42 (47.7)
Divorced	0	1 (7.7)	0	0	1 (5.6)	0	2 (2.3)
Common law	1 (7.1)	2 (15.4)	2 (10)	4 (36.4)	4 (22.2)	1 (8.3)	14 (15.9)
Widow	0	1 (7.7)	0	0	0	0	1 (1.1)
**Education *n* (%)**							
Elementary school	0	1 (7.7)	0	0	0	0	1 (1.1)
Middle school	0	1 (7.7)	0	0	0	1 (8.3)	2 (2.3)
High school	0	0	3 (15)	0	1 (5.6)	2 (16.7)	6 (6.8)
Bachelor’s degree	8 (57.2)	11 (84.6)	15 (75)	9 (81.8)	15 (83.3)	3 (25)	61 (69.3)
Technical major	3 (21.4)	0	0	1 (9.1)	0	0	4 (6.6)
Post graduated	3 (21.4)	0	2 (10)	1 (9.1)	2 (11.1)	6 (50)	14 (15.9)
**Position *n* (%)**							
Physician	2 (14.3)	4 (30.8)	4 (20)	3 (27.3)	12 (66.7)	1 (8.3)	26 (29.5)
Nurse	7 (50)	5 (38.5)	11 (55)	7 (63.6)	5 (27.8)	3 (25)	38 (43.2)
Auxiliary nurse	1 (7.1)	0	3 (15)	0	0	0	4 (4.6)
Nutritionist	4 (28.6)	2 (15.4)	2 (10)	1 (9.1)	1 (5.6)	7 (58.3)	17 (19.3)
Social worker	0	0	0	0	0	1 (8.3)	1 (1.1)
No answer	0	2 (15.4)	0	0	0	0	2 (2.3)
**Ethnicity *n* (%)**							
No	11 (78.6)	11 (84.6)	17 (85)	8 (72.7)	14 (77.8)	11 (91.7)	72 (81.8)
Yes	3 (21.4)	2 (15.4)	3 (15.0)	3 (27.3)	4 (22.2)	1 (8.3)	16 (18.2)

**Table 3:** Sociodemographic characteristics of users.

	Chiapas *n* = 19	Chihuahua *n* = 22	State of México *n* = 13	Oaxaca *n* = 16	Veracruz *n* = 24	Yucatán *n* = 30	Total *n* = 124
**Age (years)**							
Media (DE)	29 (6.4)	28.2 (6.1)	25.5 (5.2)	29 (4.8)	28.8 (6.9)	27.3 (8.5)	28.1 (6.7)
**Sex *n* (%)**							
Woman	18 (94.7)	22 (100)	12 (92.3)	16 (100)	24 (100)	29 (96.7)	121 (97.6)
Man	1 (5.3)	0	1 (7.7)	0	0	1 (3.3)	3 (2.4)
**Civil status *n* (%)**							
Single	0	2 (9.1)	0	1 (6.2)	3 (12.5)	2 (6.7)	8 (5.5)
Married	8 (42.1)	9 (40.9)	3 (23.1)	6 (37.5)	11 (45.8)	18 (60)	55 (44.4)
Divorced	0	1 (4.6)	0	0	0	0	1 (0.8)
Common law	11 (57.9)	10 (45.4)	10 (76.9)	9 (56.3)	9 (37.5)	10 (33.3)	59 (47.6)
Widow	0	0	0	0	1 (4.2)	0	1 (0.8)
**Education *n* (%)**							
None	0	1 (4.6)	0	0	0	0	1 (0.8)
Elementary school	1 (5.3)	5 (22.7)	2 (15.4)	3 (18.8)	4 (16.7)	5 (16.7)	20 (16.1)
Middle school	6 (31.6)	8 (36.4)	8 (61.5)	8 (50.0)	9 (37.5)	18 (60)	57 (46)
High school	6 (31.6)	3 (13.6)	3 (23.1)	3 (18.8)	8 (33.3)	7 (23.3)	30 (24.2)
Bachelor’s degree	4 (21.2)	5 (22.7)	0	2 (12.5)	2 (8.3)	0	13 (10.5)
Technical major	1 (5.3)	0	0	0	0	0	1 (0.8)
Post graduated	1 (5.3)	0	0	0	1 (4.2)	0	2 (1.6)
**Number of children *n* (%)**							
One	1 (5.3)	1 (4.6)	4 (30.8)	0	4 (16.7)	2 (6.7)	12 (9.7)
2 to 3	5 (26.3)	8 (36.4)	3 (23.1)	6 (37.5)	8 (33.3)	11 (36.7)	41 (33.1)
Up to 3	12 (63.2)	12 (54.5)	5 (38.5)	7 (43.8)	11 (45.8)	10 (33.3)	57 (45.9)
No answer	1 (5.2)	1 (4.5)	1 (7.7)	3 (18.8)	1 (4.2)	7 (23.3)	14 (11.3)
**Ethnicity *n* (%)**							
No	16 (84.2)	18 (81.8)	6 (46.2)	12 (75.0)	13 (54.2)	19 (63.3)	84 (67.7)
Yes	3 (15.8)	4 (18.2)	7 (53.8)	4 (25.0)	11 (45.8)	11 (36.7)	40 (32.3)

### 3.1 Contextual barriers

Low utilization to health centers and incentives to attend. The disappearance of the Social Inclusion Program (PROSPERA, as per the acronym in Spanish) had an impact on attendance at health centers, and it has affected aspects related to BF. For example, women who had a pregnancy while PROSPERA was implemented had to attend workshops where they were provided with guidance on various topics about the stage they were at, including breast milk. Those workshops are no longer implemented. Even when they had difficulties describing the information in detail, they said it was clear and useful, and they also considered that the workshops should return as a BF promotion strategy.

Sanitary emergency: the COVID-19 pandemic in 2020–2021 also affected general attendance, except for pregnant women. So, they got the regular BF’s attention. The health emergency also changed the way in which some women would get information, especially after birth, replacing control and postpartum visits to the doctor with social networks or researching on the internet.

#### 3.1.1 Lack of resources to promote BF (materials and humans)

Material resources: a systemic barrier identified was the need for more informational material (such as brochures and posters) on BF, both in Spanish and in indigenous languages. Most interviewed women mentioned that they had never been given any printed material about BF. The HP considers that it would be helpful if women could take this information with them because, they say, sometimes they do not pay attention, forget what they are told, or leave with doubts that they do not clarify. Women think having materials for later consultation at home would be useful.

One pregnant woman from Chihuahua said that “the doctor has only given me the brochures” but no specific guidance on, for example, how long she should exclusively breastfeed. In this case, the material has not made any difference since the interviewee only receives it and does not ask questions. No woman reported having observed the posters placed in some health centers, sometimes as official material from the Ministry of Health and, most of the time, prepared by nursing staff.

Human resources: those who could offer more and better BF nutritional guidance are nutrition professionals, but they are the scarcest personnel in the PHC units. Only 21 of the 95 health centers were evaluated in this study; they were nutritionists with permanent HP. In some cases, nutrition professionals assist weekly or biweekly to offer outpatient services.

Health system organization: another relevant obstacle for the population to receive preventive information from nutrition personnel is that physicians must refer them, but this is only possible when there is already a diagnosis of a poor nutrition condition. The above limits the possibilities of guiding and promoting BF to most of the population.

### 3.2 BF at primary health care: users’ perspective

#### 3.2.1 Lack of promotion at the health center

The stage of life in which the promotion of BF at the health centers most frequently occurred was pregnancy. This general information seems to focus on the baby’s benefits. Yucatán has a nutrition office, which seems to contribute to better BF promotion. The preconception women interviewed had not received any BF promotion. Some information was provided in the immediate postpartum and child stages.

*Well, the nurse just asked me if I was going to breastfeed. I not only said I would but also that I had breastfed my first daughter, so I would give this one, too. And she told me that it was healthier for the baby than formula. I think that’s all she told me* (Pregnant woman, Veracruz, rural health center).

*Yes, they gave me a talk about how to take care of the baby in what position you should put him because there are babies who settle into different positions to drink milk, so they gave us the cradle technique; when they turn upside down, they also told us they gave the twins technique, for when moms have twins. How you have to breastfeed them and all that* (Pregnant woman, Yucatán, rural health center).

Most women recognized breast milk as the first form of food that a baby should get, and formula was considered the second-best option. However, in this group of women of all life stages included in this study, the majority have used the formula. The main benefit of breast milk they mention is that “children grow up healthy.” The biggest detriment of breast milk substitutes is that “children get fat.” In addition, they realize that the formula represents an economic expense for the families. In general, they do not identify benefits for themselves. It is important to mention that it could be obtained by previous experience, through family or the internet, and not at the health center.

Pregnant women who received or found some information about BF are convinced to give breast milk to the expected child due to the benefits it offers. They get the information mainly from either their nurse or physician. The minimum time range that they would like to do it is from 3 to 6 months, but there are also several mentions, such as “*until my baby wants to drink it”* or *“until he/she is 2 years old*.” Mothers of children in the infant or preschool stage also refer to having breastfed for 6 months, some of them exclusively and some others using formula too. The reasons to stop doing it were that the baby did not want any more breast milk or that they had to work or study.

*I did breastfeed her exclusively for 6 months; then I combined it with formula because at work, they almost didn’t let me go out to feed her. Now (9 months), I have taken her off breast milk to give her a bottle. I buy the formula myself. It is very expensive. Sometimes, when I don’t have money, I make him maseca atole (a corn flour drink) with sugar.* (Mother of a child in infancy stage, Chihuahua, rural CAAPS).

*I read on verified (internet) pages that when breastfeeding, antibodies are passed to the baby that will be of use to them during their first years of life. I also asked the doctor, but he gave me little information. So, throughout my pregnancy, I was taking vitamins, and then I continued taking them, so that my baby did not lack anything in terms of nutrition and did not get sick all the time (…) I wanted to give him only breastfeeding, but in the end, I had to give him formula because when he was 1 month old, I used to go to school and I would only give him my milk* (Mother of a preschool child, Yucatan, urban health center).

PHC units are not recognized as a source for the promotion of breastfeeding. Most of the interviewees in the postpartum, child, and preschool stages reported that it was at the secondary care hospitals where they gave birth when they were informed about techniques for effective latch-on, but one could detail what she was told. There was only one case about the breast massage technique. Although she was appreciated for getting her mother’s main guidance and support.

*Yes, in the hospital, they told me that I had to breastfeed him and not to give him formula because the breast is better (…); they said to me that my baby had to latch on from the top of the nipple to be able to be suckled well, to make sure that his mouth did not sound and that he was not sucking air* (Postpartum woman, Veracruz, urban health center).

*Before I was discharged from the hospital, they gave me the information about BF printed on paper, and they explained to me how I should breastfeed my baby, how long, and how often I had to give him my breast milk.* (Mother of infant, Estado de México, rural health center).

*In the hospital, they gave me a syringe to stimulate my breast, and they barely told me about how to give myself massages. But the one who really supported me in that part was my mother, who was implementing a lot of the breast pump. We implemented the nipple cover to see if it would help him to be suckled a little more, and then we were massaging him, and all that was what helped me little by little so that she could reach the point of … well, yes, adjusting it so that he had a better grip and could suck better* (Postpartum woman, Yucatan, urban health center).

In the few cases in which women go to the nutritionist in childhood, there does not seem to be specific care for BF since the person who has been referred is the child, not the mother. However, the mothers indicated that they received the general recommendation to continue breastfeeding, and if they reported having low milk production, the advice is to drink plenty of water in order to increase it.

An interesting finding was that in rural women’s areas, the idea of breast milk losing its nutrition properties after 3 to 6 months seems to persist, so continued BF is a practice that can be jeopardized.

*I am almost going to take him off breast milk in 2 months, when he is 9 months old, because it is no longer giving him any benefit; I think that at 6 or 7 months, it is no longer beneficial. Then I will start giving him formula milk* (Mother of an infant, Chiapas, rural health center).

*I am only going to breastfeed him until he is 7 months old because the doctor tells me that at 6 months, he has had enough breast milk, and if I continue to give him milk, I can delay his learning* (Mother of Infant, State of Mexico, rural health center).

In summary, even if women do not explicitly state it, it is clear there is weak BF promotion through all stages (preconception, pregnancy, and postpartum). It seems that pregnancy is the only moment in which BF is promoted, but not enough for this practice to be carried out in later stages.

#### 3.2.2 Lack of follow-up and orientation

The length of BF is related to contextual aspects such as customs and habits or the economy, but mainly to the consultation given at the health center after the birth. Only 25% of the interviewees in the postpartum and child stages said they had exclusively breastfed for the baby’s first 6 months. The rest was combined with formula, either since birth or during some of these months. The proportion of those who continued breastfeeding without formula is even lower.

Several of the postpartum, child, and preschool stages interviewees reported using formula at least once because they could not breastfeed immediately due to actual or perceived low production or technique difficulties, so the baby was fed with it at the hospital; fortunately, in most cases, the mothers did not continue with this type of food and breastfed their babies.

*I am combining breastfeeding with formula because I am not able to get my son satisfied. They haven’t told me why I can’t fill him up. Still, the doctor has recommended that if I give him one ounce of formula now, I should give him two ounces the following month so he doesn’t stay hungry* (Postpartum woman, State of Mexico, rural Advanced Center for Primary Health Care).

*Yes, they gave my baby formula at the hospital; they offered him formula on one occasion just because he could not suckle my breast well. But now I only give him my milk* (Postpartum Woman, Oaxaca, rural health center).

It was mentioned that pregnant women get basic information about BF. However, when the time to breastfeed comes, if they do not receive guidance in the face of any difficulty or doubt, they will likely stop giving breast milk. None of the women interviewed received guidance on latching techniques, milk extraction, or breast massaging during the puerperium.

As mentioned, postpartum women do not attend check-ups unless they have an alert sign. In this sense, it seems that BF difficulties are not recognized as relevant and require immediate attention. Although attendance is higher in childhood, according to the mothers interviewed, no special attention is given to BF. During control visits, some nurses and doctors ask if they are sharing breast milk. They continue the review without delving into details if the answer is positive. If they comment that they are having difficulties, few nurses offer advice on latching techniques and breast massaging, but doctors directly recommend introducing formula. Women follow this instruction because it relieves their anxiety about not “filling” (satisfying their babies). Although most of them express remorse because they know or “feel” that they are denying a considerable benefit to their children, there were also cases of women who, despite receiving the recommendation from their physician or nutritionist to give Exclusive BF for up to 6 months and continued for up to 2 years, decided to stop BF when they returned to work because they did not have the time and conditions to extract breast milk or because it was too difficult for them to get their job done and feed their baby from the breast.

*The doctor tells me to take him off the formula and give him only breast milk, but I tell my husband that I can’t takes it away from my baby because otherwise, he won’t be satisfied. The doctor told me he is fine, but I should give him formula once a day and my milk twice a day because he is growing very fast. And he is fine, but as I told you, I work in a store, and I get tired, and since my husband works at night, there is no one to help me, I am the only one who gets up at night. I don’t rest well, that’s why I want to take it away from him* (Mother of a child in infancy, Yucatan, rural health center).

### 3.3 Barriers from health professionals’ perspective

#### 3.3.1 Not enough time, not enough knowledge

A systemic barrier is the time HP has for each patient: 15 min on average. Physicians and most nutritionists think it is enough time. Still, nurses consider that it is not enough to provide them with all the information and, at the same time, be able to clear up their doubts, especially for first-time pregnant women.

Health personnel recognize BF as a very important practice for the child’s health. However, when describing what a controlled medical consultation is like, only six of the interviewed physicians mentioned the promotion of BF as part of this care. Nutritionists do not include it because they mostly care for patients referred for a specific situation identified by the doctor. The nurses are shown as the ones who do the most promotion, some during the brief nutritional control procedure and others trying to give talks to groups of pregnant women. Three nurses mentioned specific cases in which they have guided mothers of children in infancy, recommending grasping techniques and breast massages to promote production.

*Sometimes if the mother brings her 6 months-old child who already has to start weaning, I have to tell the mother “No, you have to give him this now; it is no longer exclusive breastfeeding.” But, if they don’t bring him and the mother only continues to breastfeed him, and she doesn’t give him (complementary) food, the child will fall into malnutrition. So, if they don’t bring the child, well … We don’t guide them on these issues*—Nurse, Chiapas, rural.

The medical and nursing staff acknowledged having little knowledge about nutrition and BF. So, when they promote it over follow-up visits during pregnancy, they mention basic and common-sense aspects such as “it is good for healthy growth.” No HP mentions the guidance provided regarding breast massage or milk extraction at any stage.

#### 3.3.2 Low users’ attendance and no attending

In general, HP reported providing BF information during pregnancy because they knew it was very important. They are unsure if the information is clear enough to women because, although they do not regularly ask them questions or express doubts, they have identified that they pay little or no attention to the recommendations they generally make. So they may not follow the guidance provided.

In relation to women in preconception, HP points out that they are not users who come due to a lack of a culture of preparation for pregnancy; anyway—or because of that—it seems that they do not have a specific strategy to promote BF if any woman attends prenatal controls. The actions related to BF taken by the HP occur mainly during pregnancy, and they hope (or expect) that this will be sufficient to ensure that women do not have barriers to breastfeeding their children. For example, in one state, dolls are used to teach latching techniques to pregnant women, but not during puerperium.

*Breastfeeding is one of our pillars, one of our strongest actions. We have materials such as the “Jorgitos” (dolls) to teach them latching techniques and the “mama breasts” to teach them how to get milk; in other words, there are some resources to work with pregnant women.* (Nutritionist, Yucatan, urban).

*During pregnancy-control-consultation, breastfeeding is explained, as well as its importance, the complications that may occur if nothing is done, the use of bottles, and its complications. If she comes and is already breastfeeding, she is oriented on what to do after breastfeeding, on her digestion, on the use of the straw. These little details take time, but women need this orientation* (Physician, Veracruz, Urban Health Center).

According to HP, women in the postpartum period usually do not attend medical consultations unless they have some warning signs. This explains why only 10 interviews and no focus groups with this profile were achieved. Therefore, they generally do not receive any guidance on effective latch-on techniques, breast massaging, or milk extraction at the health center during this period, without mentioning the lack of trained personnel who could provide this information.

In the case of children under 2 years of age, the health unit attendance was higher; however, the main reason for attendance was the vaccine administration and not a control consultation unless the child had a health problem. Some nurses recognized that vaccine administration visits could be used to provide nutritional guidance, including BF. Still, they do not have enough time to vaccinate and comply with the administrative process that it implies.

*When I apply the vaccines to a child, I would like to do more, check how they are in weight and height, and monitor how their nutrition is going (…), but I am the only nurse here. If at a certain moment, I am vaccinating and at the same time checking one by one, I’ll be late. Plus, others are waiting for their vaccines, so sometimes time limits me. Yes, I would like to cover many things, such as guiding mothers, especially first-timers, since they may have many doubts, but the lack of time and nursing staff limits me in everything.* (Nurse, Oaxaca, rural health center).

Regarding the economic costs of commercial milk formulas, the medical position is contradictory, as they recommend using milk formula despite its high cost to the household economy. However, they also recognize that the need for more home resources is a barrier to the following nutritional recommendations.

#### 3.3.3 “The other” promotion

Physicians said they follow up on the type of BF mothers offer their children under 2 years of age, recommending exclusive breastfeeding for up to 6 months and continuing up to 2 years. However, they acknowledge that they recommend giving a formula when women report physical or contextual difficulties to provide it. Nursing and nutrition personnel seem more inclined to promote BF than physicians, showing more sensitivity and knowledge.

*We have had very, very malnourished children. That’s why I always (recommend to moms) breastfeed, latch on well. You hold your baby’s head with one hand, and with the fingers of the other hand, you have the nipple, and that’s how you’re going to feed him. And look at your baby, let your baby look at you, let him see that connection. Give that time to your baby* (Nurse, State of Mexico, rural health center).

The interviews with the HP showed that exclusive BF and continued BF were promoted in the control visits to children under 2 years of age, focusing on the benefits of this food. The medical and nursing staff mentioned that they ask the mothers if they breastfeed their babies and, unless they report any problems in doing so, they are satisfied with the answer “yes,” and no further inquiries are made. When difficulties in BF appear, which are mostly “I do not have enough milk and it does not my child satisfied” or “the baby does not like my milk,” the nurses make recommendations such as drinking plenty of water and giving breast massages or “home remedies” such as drinking *atole*. (A corn flour drink).

Nutrition personnel are the most reluctant to recommend milk formula and do so only when the child or mother is diagnosed with malnutrition. However, because their attention depends on the physician’s decision to refer the patient to the nutrition service, the promotion of BF is limited. A case was found in which the breast milk substitute industry gives away unique formulas to mothers through a nutritionist who recognizes that there is a commercial strategy for the company but considers that it is a way to support children with special feeding needs.

*This formula is for children who do not tolerate regular milk. It is a special milk for children who become constipated and have no infant formula left. This line has one for children under one year old, and this one is for children from one to three years old. We are supported by the Nestlé promoter, who provides us with courses and gives us material and substitutes. We ask her specifically for the children we detect with some pathology, and she brings them to us so that the mother can try it and repurchase it. I know that the company does this to sell more … and yes, it is expensive, about $250 a can* (Nutritionist, Veracruz, urban health center).

**Figure 1:**
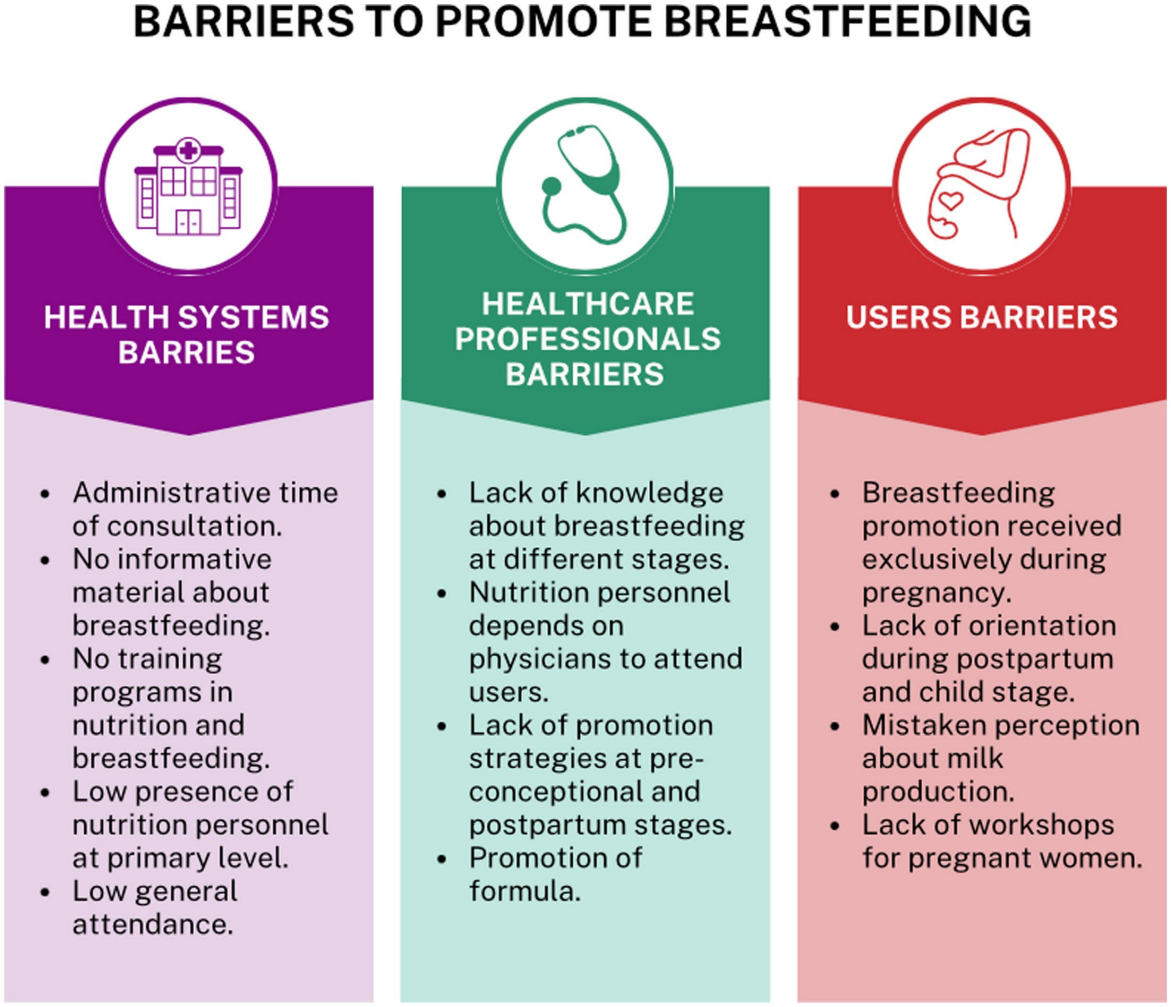
Barriers to promote breastfeeding in primary health care in Mexico.

Figure 1, shown a synthesis of the barriers to promote breastfeeding in PHC in Mexico.

## 4 Discussion

In Mexico, there are still barriers to the promotion of breastfeeding for mothers who receive care in public health services. The disappearance of the PROSPERA, the interruption in the continuity of care for pregnant women, the shortage of nutrition professionals within the units, the lack of promotion of breastfeeding from preconception, the recommendation of milk formulas as a source of food, the existence of myths about the quality of breast milk, the lack of material that promotes BF and that is not adapted to the sociocultural contexts of each region, and the lack of reinforcement of breastfeeding during newborn control were the main barriers identified from the perspective of HP and users for the promotion of BF.

The main findings related to the interruption were the disappearance of PROSPERA and the interruption in the continuity of care during the COVID-19 epidemic. This is related to the evaluated years in the study (2020 and 2021); access to and delivery of health services for the population without social security were affected by two reasons. The first was due to mobility restrictions and the saturation of health units due to the SARS-CoV-2 epidemic (COVID-19). This means a reduction in the number of consultations, restricted mobility, and fear of contagion within the health units (31, 34). The second one was the transition that the public health system was going through, repealing the Social Protection System in Health and replacing it with INSABI. In this transition, the HP reported that the decline in control attendance, especially of the “healthy child,” began when the health component of the conditional cash transfer program, known as PROSPERA, was canceled in 2019. This program covered 6.5 million households and the disappearance of the co-responsibility of attending control appointments in exchange for receiving economic support (35, 36).

It is evident that women’s interest in BF their children, but the limited promotion it gets during pregnancy and the practically non-existent orientation in the postpartum and childhood stages discourage this objective. The idea of low milk production persists in women, and HP reaffirms it even more. The solution that physicians suggest for this and some other difficulties is the use of formula, especially when women feel guilty about not satisfying their children with enough food. These findings show the unfamiliarity and persistent violation of the International Code of Marketing of Breastmilk Substitutes by HP, phenomena previously addressed by other authors in the Mexican context (37, 38).

Counseling on effective latch-on techniques, breast massaging, and milk extraction hardly occurs during the postpartum period because women at this stage usually do not come to the health center. However, it is also considered that there is a well-designed strategy or guidelines to guide the few postpartum women who come for follow-up. Our study agrees with the results published by Hernández Cordero et al. (39), which showed that one of the barriers to breastfeeding identified by the Mexican Health System is the lack of advice from health personnel.

The intervention of more qualified personnel in the nutritional field could counteract this problem; however, nutritionists are the ones who need more presence in the PHCs. On the other hand, nursing staff could also be key in the promotion and orientation of BF since they are more sensitized; most of them do not have sufficient or updated training, and a significant administrative burden keeps them from spending more time on other topics. This reflects the need to strengthen the PHC system with more trained maternal and child nutrition professionals.

Some positive aspects were found within the units that provide care to the population without social security, such as the existence of a Nutrition Directorate in the state of Yucatan. This direction favors the availability of nutrition professionals; however, the overall results show a lack of promotion and counseling of BF. In the case of Chihuahua, some health centers reported that the nutrition professional visits the health center once every 15 days or once a month. This situation also acts as a barrier to the control and monitoring of users (40).

The data collection period was also identified as a limitation for collecting information since we consider that the COVID-19 pandemic was a period of stress, social distancing, and difficulties in accessing health services, mainly by the users. Our study shows the barriers to adequate breastfeeding practices in the selected states of Mexico; however, it is necessary to have more studies in other regions of the country to have a complete vision of the problem.

In conclusion, there are barriers to breastfeeding counseling and promotion in first-level units in six states. Although there are strategies for the promotion of exclusive and continuous breastfeeding at the first level of care, there are still contextual barriers that prevent women from receiving this promotion. This study shows the need to train PSs at the first level of care on issues of breastfeeding and lactation for postpartum mothers, counseling these messages during prenatal visits. Similarly, it is important to create programs or strategies that allow the control of children’s health in PHC to delay the different forms of malnutrition.

Considering the results, we have identified the urgent need to implement actions aimed at improving the quality of nutritional care in PHC on breastfeeding issues. These actions can have a significant impact on optimizing the nutritional status of the maternal-infant population and influencing the prevention of intergenerational transmission of pathological conditions and cardiometabolic risks during the life course.

## Data availability statement

The raw data supporting the conclusions of this article will be made available by the authors, without undue reservation.

## Ethics statement

The studies involving humans were approved by the Ethics Committee of the Universidad Iberoamericana in Mexico City (No. 103/2021) and it was authorized by the Ministry of Health of each state. The studies were conducted in accordance with the local legislation and institutional requirements. The participants provided their written informed consent to participate in this study.

## Author contributions

EH-L: Formal analysis, Investigation, Methodology, Supervision, Writing – original draft. CPN: Conceptualization, Data curation, Formal analysis, Investigation, Methodology, Supervision, Writing – original draft. SB-M: Supervision, Writing – review & editing. SH-C: Supervision, Writing – review & editing. IO-G: Supervision, Writing – review & editing. MAA: Supervision, Writing – review & editing. MA-M: Conceptualization, Data curation, Funding acquisition, Methodology, Project administration, Supervision, Validation, Visualization, Writing – original draft.
