# Corrigendum: Barriers to promoting breastfeeding in primary health care in Mexico: a qualitative perspective

**DOI:** 10.3389/fnut.2024.1372964

**Published:** 2024-02-29

**Authors:** Elizabeth Hoyos-Loya, Cecilia Pérez Navarro, Soraya Burrola-Méndez, Sonia Hernández-Cordero, Isabel Omaña-Guzmán, Matthias Sachse Aguilera, Mónica Ancira-Moreno

**Affiliations:** ^1^Observatorio Materno Infantil (OMI), Universidad Iberoamericana, Mexico City, Mexico; ^2^Health Department, Universidad Iberoamericana, Mexico City, Mexico; ^3^Research Center for Equitable Development EQUIDE, Universidad Iberoamericana, Mexico City, Mexico; ^4^Pediatric Obesity Clinic and Wellness Unit, Hospital General de México “Dr. Eduardo Liceaga”, Mexico City, Mexico; ^5^United Nations International Children's Emergency Fund (UNICEF), Mexico City, Mexico

**Keywords:** breastfeeding, primary health care, child health services, quality of health care, postpartum, infant, prenatal care

In the published article, there was an error in Table 1 as published. The mentions column should not have been included. The corrected Table 1 and its caption appear below.

**Table 1:** Breastfeeding codebook.

**Actor**	**Category**	**Description**
User	BF knowledge and Information	Overview about the users' knowledge and how they get it
	BF recommendations	Guidance on techniques for effective latching, breast extraction, breast massage users receive at the health center
	BF length	Period that women say they have given exclusive breastfeeding and/or continued breastfeeding
	BF barriers	Difficulties identified by women to follow the BF recommendations during the postpartum period, the infant and the preschool child stage
	Formula	Use or introduction of milk formula in babies reported by women
Health professionals	BF promotion	Strategies or actions to promote exclusive breastfeeding and continued breastfeeding carried out by the HP in the health center
	BF guidance	Breastfeeding information HP give to users during control medical consultation
	BF follow-up	Follow-up given to breastfeeding provided by postpartum mothers and up to 2 years of age of the child
	BF barriers	Difficulties identified by HP for women to follow the BF guidance or recommendations

BF, breastfeeding.

In the published article, there was an error, the “Ministry of Health in Mexico” was inadvertently duplicated.

A correction has been made to Abstract, methodology, Paragraph 1. This sentence previously stated:

“A qualitative study with a phenomenological approach was carried out in 88 health centers of the Ministry of Health in the states of Chihuahua, Oaxaca, Chiapas, Veracruz, Mexico, and Yucatan. From September to November 2021, we interviewed 88 key health professionals (HPs) (physicians, nurses, nutritionists, and others) from the PHC of the Ministry of Health in Mexico and 80 parents of children under 5 years old. In addition, nine focus groups were conducted with parents and caregivers. The data obtained were triangulated with information from focus groups and semi-structured interviews.”

The corrected sentence appears below:

“A qualitative study with a phenomenological approach was carried out in 88 health centers of the Ministry of Health in the states of Chihuahua, Oaxaca, Chiapas, Veracruz, Mexico, and Yucatan. From September to November 2021, we interviewed 88 key health professionals (HPs) (physicians, nurses, nutritionists, and others) from the PHC and 80 parents of children under 5 years old. In addition, nine focus groups were conducted with parents and caregivers. The data obtained were triangulated with information from focus groups and semi-structured interviews.”

In the published article, there was an error, “(n=97)” was repeated.

A correction has been made to Materials and methods, 2.1 Population and study unit, Paragraph Number 2. This sentence previously stated:

“For the selection of user participants or their partners, the criteria included: (a) being a woman in preconception, (b) being pregnant, or (c) being a parent of a child under 5 years. Regarding HP, the criterion was initially to have worked in the health center for at least 2 years. However, due to frequent staff rotation, this criterion was eliminated. In both groups, participants needed to be at least 18 years old and provide signed informed consent to participate in the research. At least one interview with an HP (n = 97), an interview with a user (n = 97) in each health center, and 30 focus groups (5 in each state) were expected to be conducted.”

The corrected sentence appears below:

“For the selection of user participants or their partners, the criteria included: (a) being a woman in preconception, (b) being pregnant, or (c) being a parent of a child under 5 years. Regarding HP, the criterion was initially to have worked in the health center for at least 2 years. However, due to frequent staff rotation, this criterion was eliminated. In both groups, participants needed to be at least 18 years old and provide signed informed consent to participate in the research. At least one interview with an HP, an interview with a user in each health center, and 30 focus groups (5 in each state) were expected to be conducted.”

In the published article, there was an error, a section number was omitted.

A correction has been made to Results, 3.1 Contextual barriers

This section heading previously stated:

“Lack of resources to promote BF (materials and humans).”

The corrected section heading appears below:

“3.1.1 Lack of resources to promote BF (materials and humans).”

The authors apologize for these errors and state that this does not change the scientific conclusions of the article in any way. The original article has been updated.

